# Supplementary material for: Meta-Transcriptomic Analysis Uncovers the Presence of Four Novel Viruses and Multiple Known Virus Genera in a Single Hibiscus rosa-sinensis Plant in Colombia
Source: Viruses. 2024 Feb 7;16(2):267. doi: 10.3390/v16020267 (PMC10891833; doi:10.3390/v16020267)
Supplement: Supplementary file 1 [file viruses-16-00267-s001.zip › viruses-2848632-supplementary.pdf]

Supplementary Figure S1: Heatmap analysis of the pairwise amino acid identity matrixes of hibiscus carlaviruses (HiCaV-A, HiCaV-B, HiCaV-C, and HiCaV-D) infecting the RsTHr hibiscus sample. Collectively, 45, 40, and 35 carlavirus species Triple Gene Block (TGB) 1 (S1A), TGB2 (S1B), and TGB3 (S1C), amino acid sequences, respectively, were included from the NCBI database. Different shades of blue triangle for TGB1 to TGB3 inside the red-bordered rectangles refer to less than 55% amino acid identity of HiCaVs with any carlavirus sequences included in this analysis. Phylogenetic relationships of the hibiscus carlaviruses (HiCaV-A, HiCaV-B, HiCaV-C, and HiCaV-D) identified in the RsTHr high-throughput sequencing library with the TGB1 (S1D), TGB2 (S1E), and TGB3 (S1F) complete amino acid sequences of 35 to 45 *Betaflexiviridae* family members using the Multiple Alignment using Fast Fourier Transform (MAFFT, v7.520) program with bootstrap values of 1000 replicates. The names corresponding to the accession numbers in the heatmap analysis are displayed in the phylogenetic trees created using the TGB-1, -2, and -3 amino acid sequences.

(S1A)

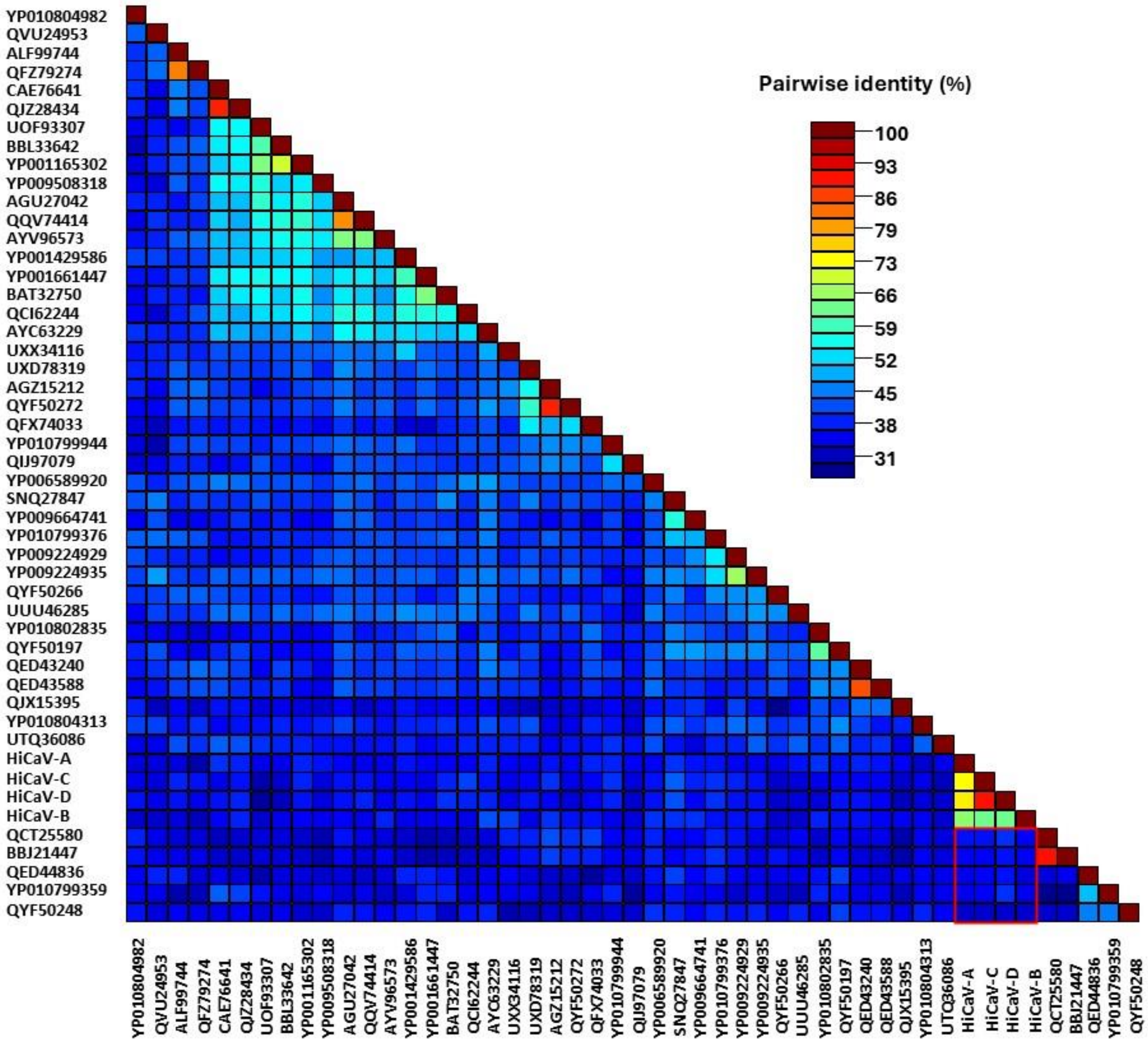

(S1B)

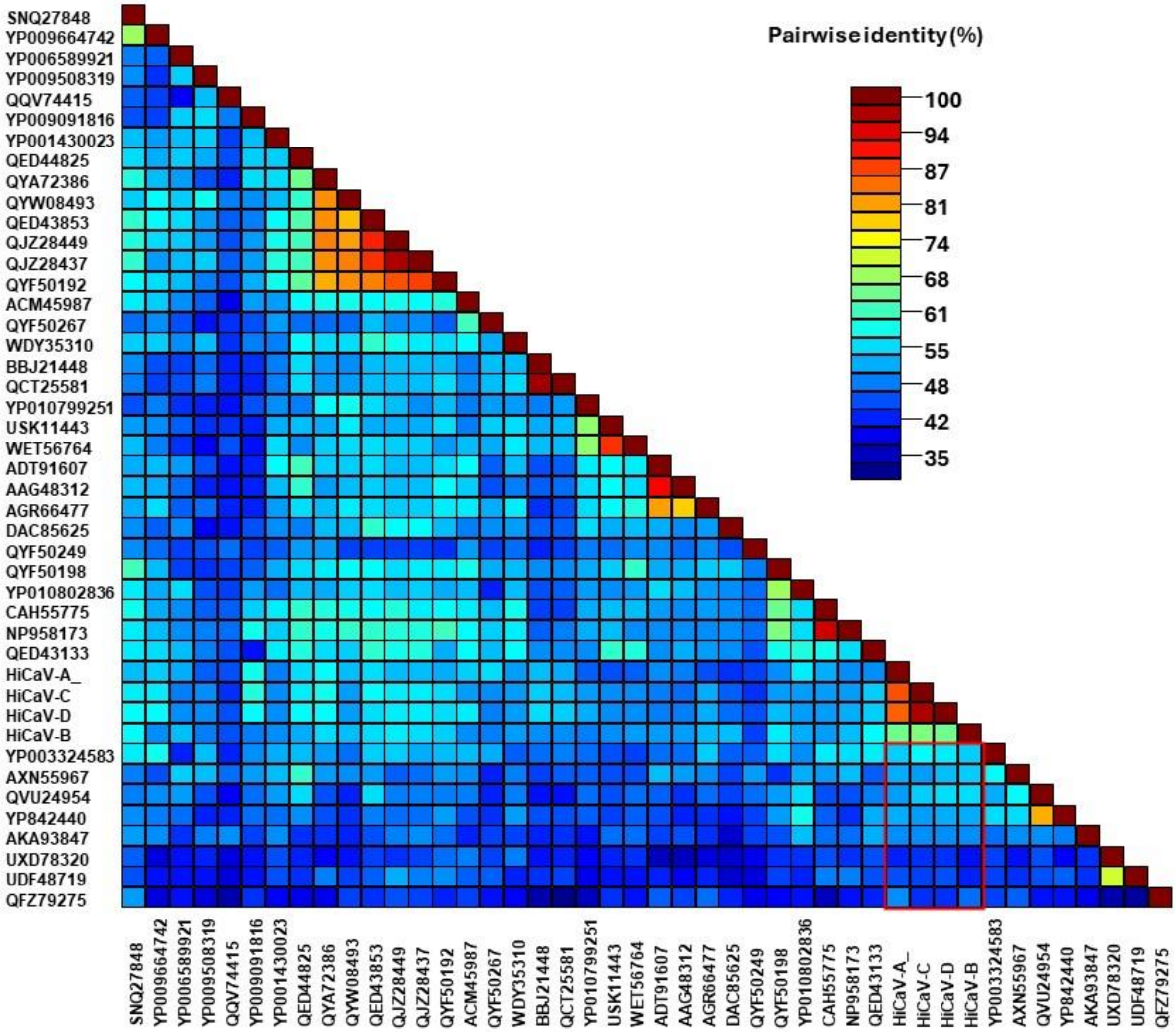
Pairwise identity (%)
100
94
87
81
74
68
61
55
48
42
35
SNQ27848
YP009664742
YP006589921
YP009508319
QQV74415
YP009091816
YP001430023
QED44825
QYA72386
QYW08493
QED43853
QJZ28449
QJZ28437
QYF50192
ACM45987
QYF50267
WDY35310
BBJ21448
QCT25581
YP010799251
USK11443
WET56764
ADT91607
AAG48312
AGR66477
DAC85625
QYF50249
QYF50198
YP010802836
CAH55775
NP958173
QED43133
HiCaV-A_
HiCaV-C
HiCaV-D
HiCaV-B
YP003324583
AXN55967
QVU24954
YP842440
AKA93847
UXD78320
UDF48719
QFZ79275

(S1C)

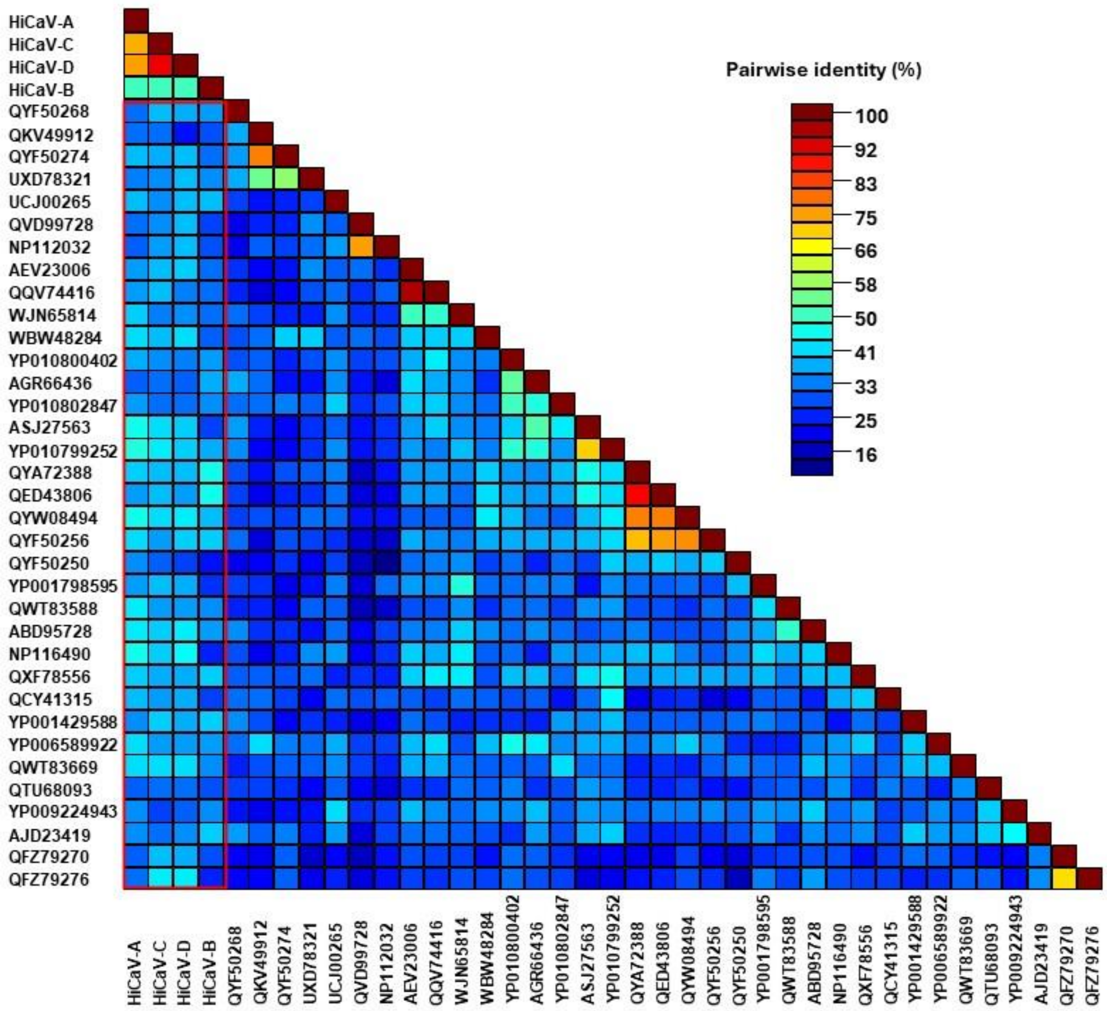
Pairwise identity (%)
100
92
83
75
66
58
50
41
33
25
16
HiCaV-A
HiCaV-C
HiCaV-D
HiCaV-B
QYF50268
QKV49912
QYF50274
UXD78321
UCJ00265
QVD99728
NP112032
AEV23006
QQV74416
WJN65814
WBW48284
YP010800402
AGR66436
YP010802847
ASJ27563
YP010799252
QYA72388
QED43806
QYW08494
QYF50256
QYF50250
YP001798595
QWT83588
ABD95728
NP116490
QXF78556
QCY41315
YP001429588
YP006589922
QWT83669
QTU68093
YP009224943
AJD23419
QFZ79270
QFZ79276

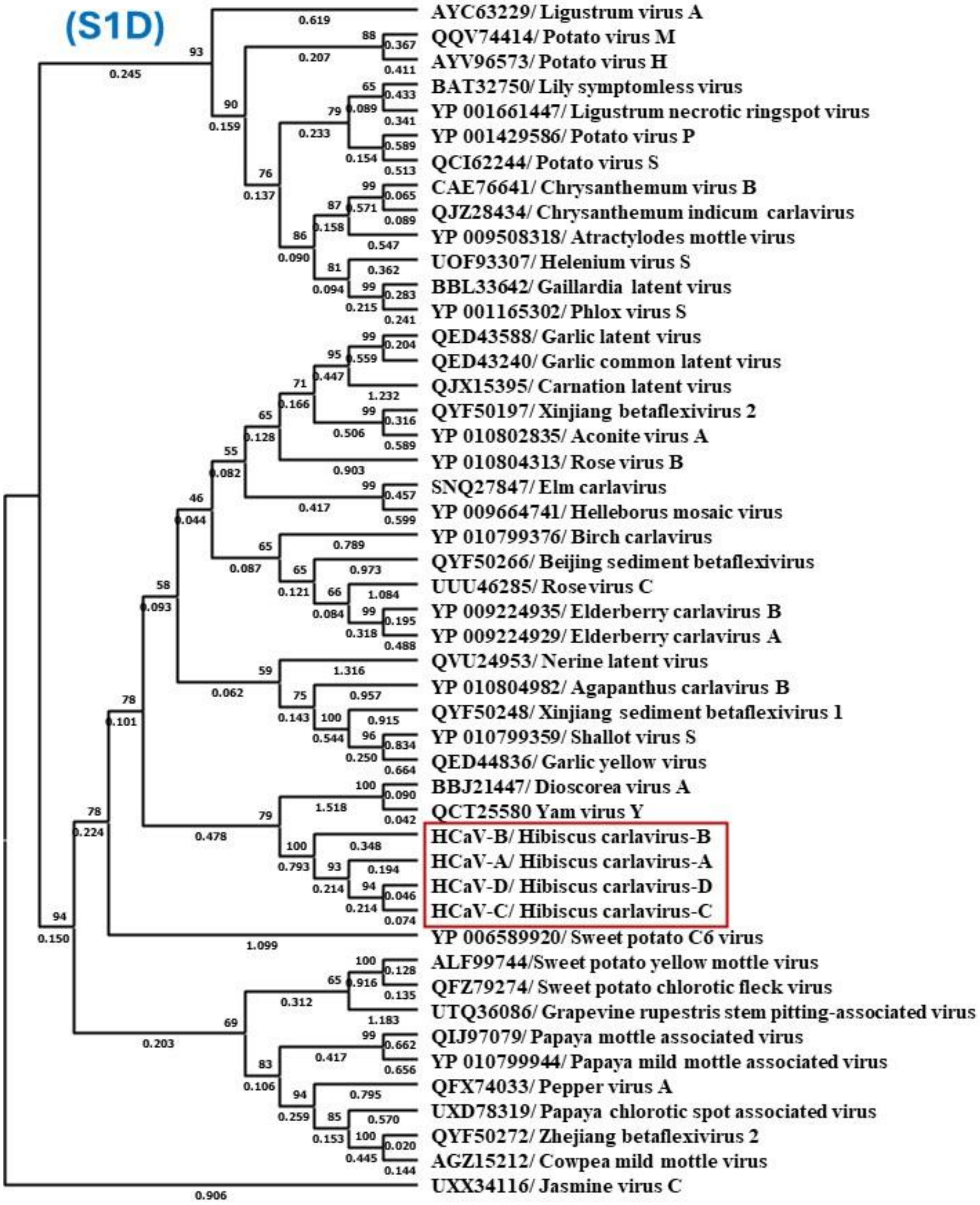
(S1D)
AYC63229/ Ligustrum virus A
QQV74414/ Potato virus M
AYV96573/ Potato virus H
BAT32750/ Lily symptomless virus
YP 001661447/ Ligustrum necrotic ringspot virus
YP 001429586/ Potato virus P
QCI62244/ Potato virus S
CAE76641/ Chrysanthemum virus B
QJZ28434/ Chrysanthemum indicum carlavirus
YP 009508318/ Atractylodes mottle virus
UOF93307/ Helenium virus S
BBL33642/ Gaillardia latent virus
YP 001165302/ Phlox virus S
QED43588/ Garlic latent virus
QED43240/ Garlic common latent virus
QJX15395/ Carnation latent virus
QYF50197/ Xinjiang betaflexivirus 2
YP 010802835/ Aconite virus A
YP 010804313/ Rose virus B
SNQ27847/ Elm carlavirus
YP 009664741/ Helleborus mosaic virus
YP 010799376/ Birch carlavirus
QYF50266/ Beijing sediment betaflexivirus
UUU46285/ Rose virus C
YP 009224935/ Elderberry carlavirus B
YP 009224929/ Elderberry carlavirus A
QVU24953/ Nerine latent virus
YP 010804982/ Agapanthus carlavirus B
QYF50248/ Xinjiang sediment betaflexivirus 1
YP 010799359/ Shallot virus S
QED44836/ Garlic yellow virus
BBJ21447/ Dioscorea virus A
QCT25580 Yam virus Y
HCaV-B/ Hibiscus carlavirus-B
HCaV-A/ Hibiscus carlavirus-A
HCaV-D/ Hibiscus carlavirus-D
HCaV-C/ Hibiscus carlavirus-C
YP 006589920/ Sweet potato C6 virus
ALF99744/Sweet potato yellow mottle virus
QFZ79274/ Sweet potato chlorotic fleck virus
UTQ36086/ Grapevine rupestris stem pitting-associated virus
QIJ97079/ Papaya mottle associated virus
YP 010799944/ Papaya mild mottle associated virus
QFX74033/ Pepper virus A
UXD78319/ Papaya chlorotic spot associated virus
QYF50272/ Zhejiang betaflexivirus 2
AGZ15212/ Cowpea mild mottle virus
UXX34116/ Jasmine virus C

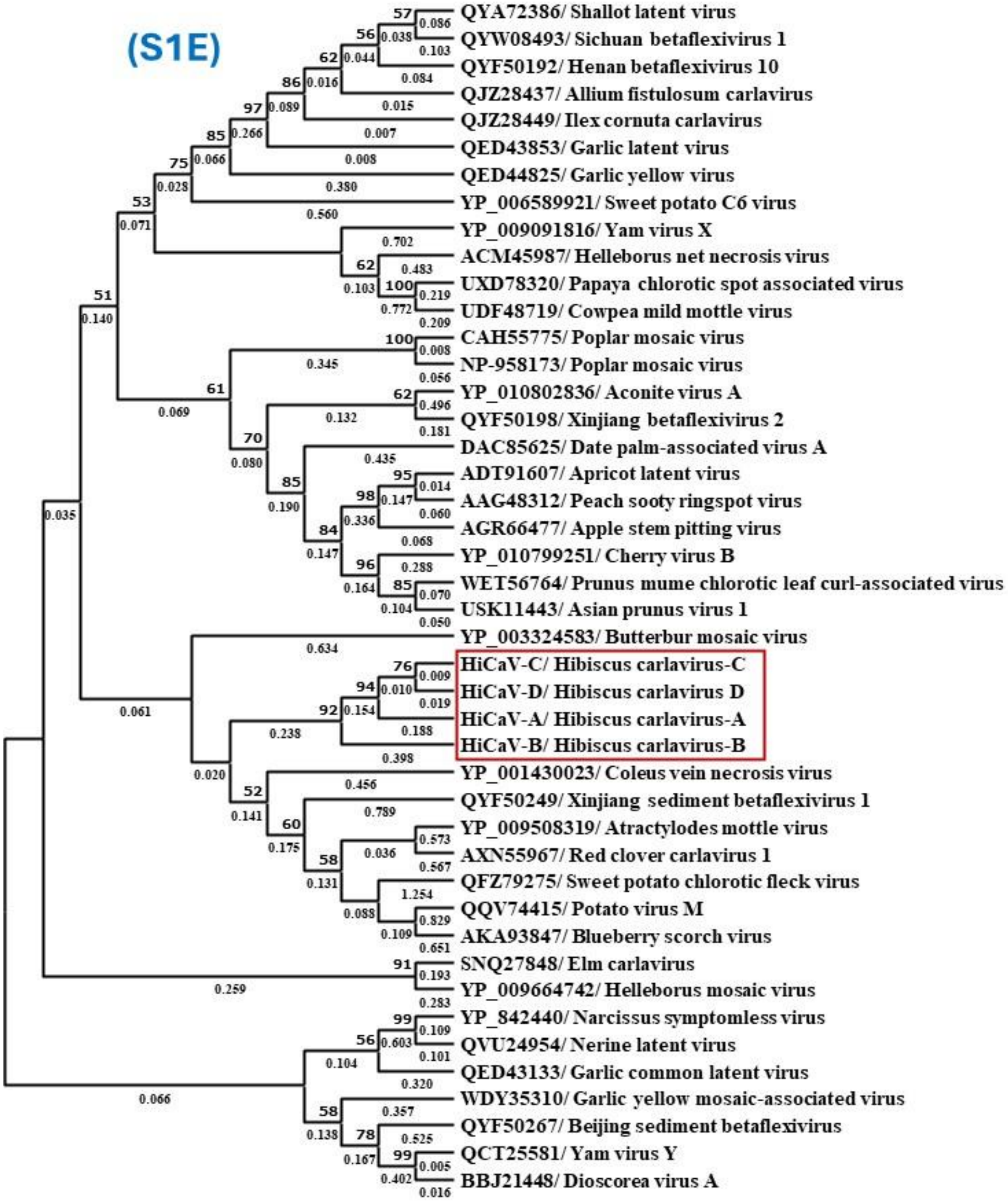
(S1E)
QYA72386/ Shallot latent virus
QYW08493/ Sichuan betaflexivirus 1
QYF50192/ Henan betaflexivirus 10
QJZ28437/ Allium fistulosum carlavirus
QJZ28449/ Ilex cornuta carlavirus
QED43853/ Garlic latent virus
QED44825/ Garlic yellow virus
YP_006589921/ Sweet potato C6 virus
YP_009091816/ Yam virus X
ACM45987/ Helleborus net necrosis virus
UXD78320/ Papaya chlorotic spot associated virus
UDF48719/ Cowpea mild mottle virus
CAH55775/ Poplar mosaic virus
NP-958173/ Poplar mosaic virus
YP_010802836/ Aconite virus A
QYF50198/ Xinjiang betaflexivirus 2
DAC85625/ Date palm-associated virus A
ADT91607/ Apricot latent virus
AAG48312/ Peach sooty ringspot virus
AGR66477/ Apple stem pitting virus
YP_010799251/ Cherry virus B
WET56764/ Prunus mume chlorotic leaf curl-associated virus
USK11443/ Asian prunus virus 1
YP_003324583/ Butterbur mosaic virus
HiCaV-C/ Hibiscus carlavirus-C
HiCaV-D/ Hibiscus carlavirus D
HiCaV-A/ Hibiscus carlavirus-A
HiCaV-B/ Hibiscus carlavirus-B
YP_001430023/ Coleus vein necrosis virus
QYF50249/ Xinjiang sediment betaflexivirus 1
YP_009508319/ Atractylodes mottle virus
AXN55967/ Red clover carlavirus 1
QFZ79275/ Sweet potato chlorotic fleck virus
QQV74415/ Potato virus M
AKA93847/ Blueberry scorch virus
SNQ27848/ Elm carlavirus
YP_009664742/ Helleborus mosaic virus
YP_842440/ Narcissus symptomless virus
QVU24954/ Nerine latent virus
QED43133/ Garlic common latent virus
WDY35310/ Garlic yellow mosaic-associated virus
QYF50267/ Beijing sediment betaflexivirus
QCT25581/ Yam virus Y
BBJ21448/ Dioscorea virus A

(S1F)

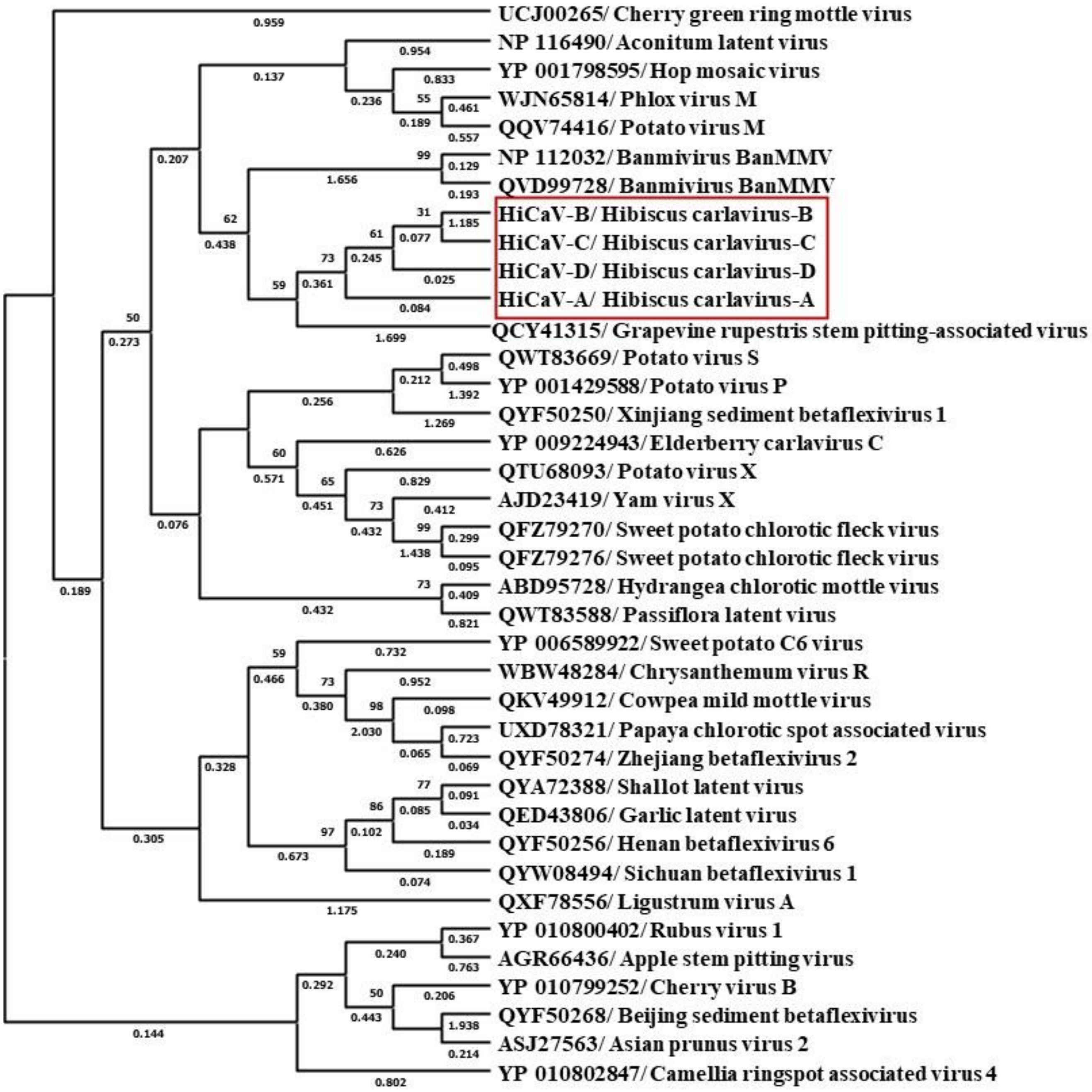
UCJ00265/ Cherry green ring mottle virus
NP 116490/ Aconitum latent virus
YP 001798595/ Hop mosaic virus
WJN65814/ Phlox virus M
QQV74416/ Potato virus M
NP 112032/ Banmivirus BanMMV
QVD99728/ Banmivirus BanMMV
HiCaV-B/ Hibiscus carlavirus-B
HiCaV-C/ Hibiscus carlavirus-C
HiCaV-D/ Hibiscus carlavirus-D
HiCaV-A/ Hibiscus carlavirus-A
QCY41315/ Grapevine rupestris stem pitting-associated virus
QWT83669/ Potato virus S
YP 001429588/ Potato virus P
QYF50250/ Xinjiang sediment betaflexivirus 1
YP 009224943/ Elderberry carlavirus C
QTU68093/ Potato virus X
AJD23419/ Yam virus X
QFZ79270/ Sweet potato chlorotic fleck virus
QFZ79276/ Sweet potato chlorotic fleck virus
ABD95728/ Hydrangea chlorotic mottle virus
QWT83588/ Passiflora latent virus
YP 006589922/ Sweet potato C6 virus
WBW48284/ Chrysanthemum virus R
QKV49912/ Cowpea mild mottle virus
UXD78321/ Papaya chlorotic spot associated virus
QYF50274/ Zhejiang betaflexivirus 2
QYA72388/ Shallot latent virus
QED43806/ Garlic latent virus
QYF50256/ Henan betaflexivirus 6
QYW08494/ Sichuan betaflexivirus 1
QXF78556/ Ligustrum virus A
YP 010800402/ Rubus virus 1
AGR66436/ Apple stem pitting virus
YP 010799252/ Cherry virus B
QYF50268/ Beijing sediment betaflexivirus
ASJ27563/ Asian prunus virus 2
YP 010802847/ Camellia ringspot associated virus 4

Supplementary Figure S2: Phylogenetic relationships of the newly discovered hibiscus potexvirus (HiVX) in the RsTHr high-throughput sequencing library with 57 potexvirus species genome sequences (at nucleotide level) using the Multiple Alignment using Fast Fourier Transform (MAFFT, v7.520) program with bootstrap values of 1000 replicates.

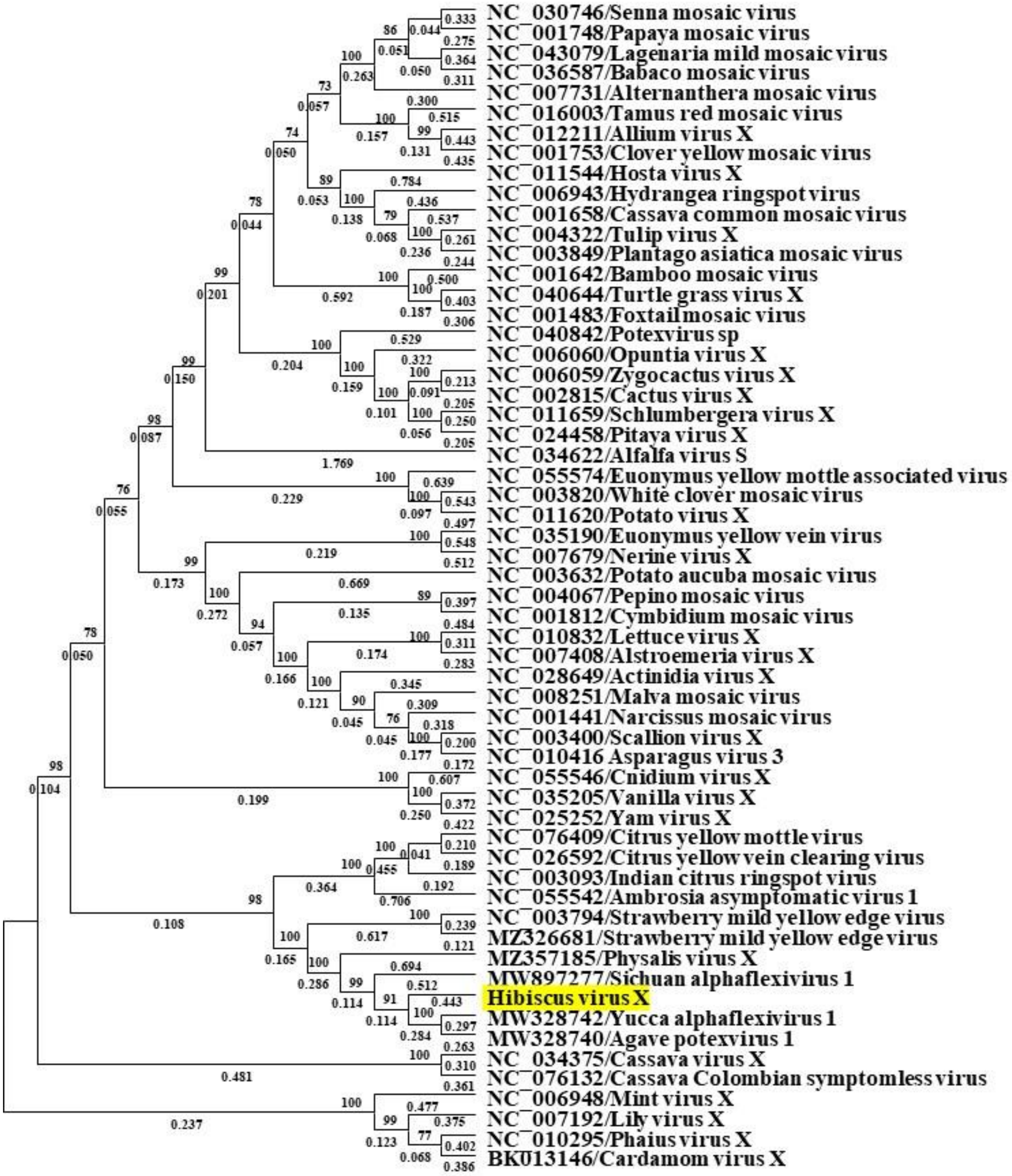

Supplementary Figure S3: Phylogenetic relationships of the newly discovered hibiscus potexvirus (HiVX) in the RsTHr high-throughput sequencing library with the most closely related potexvirus TGB1 (S3A), TGB2 (S3B) and TGB3 (S3C) amino acid sequences available in GenBank utilizing the Multiple Alignment using Fast Fourier Transform (MAFFT, v7.520) program with bootstrap values of 1000 replicates.

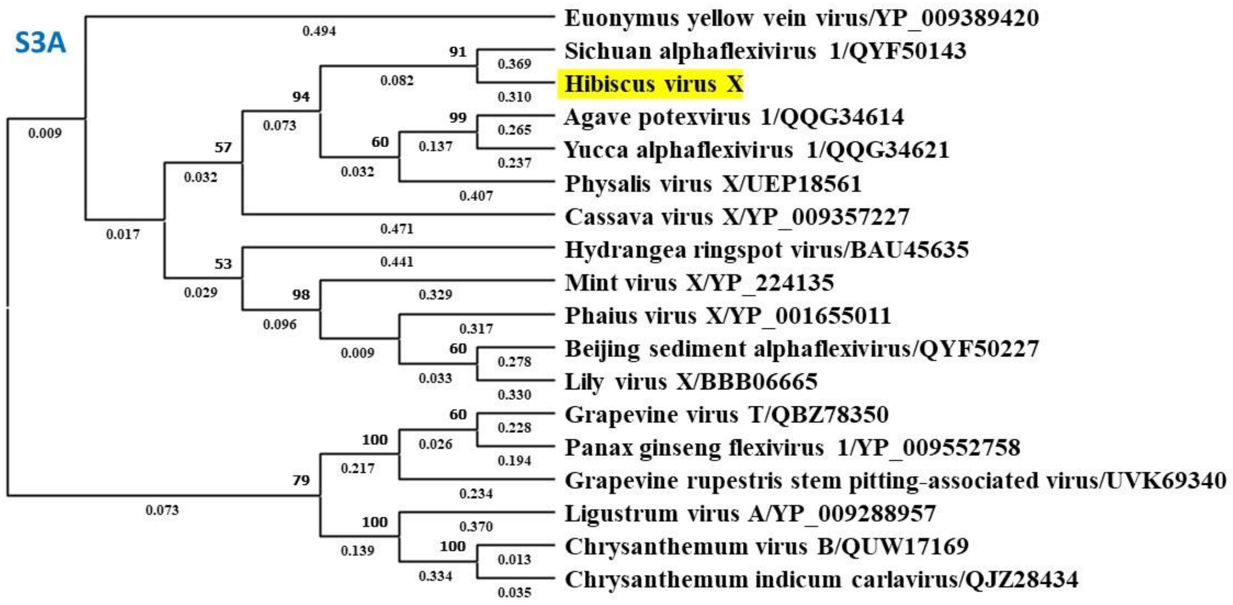

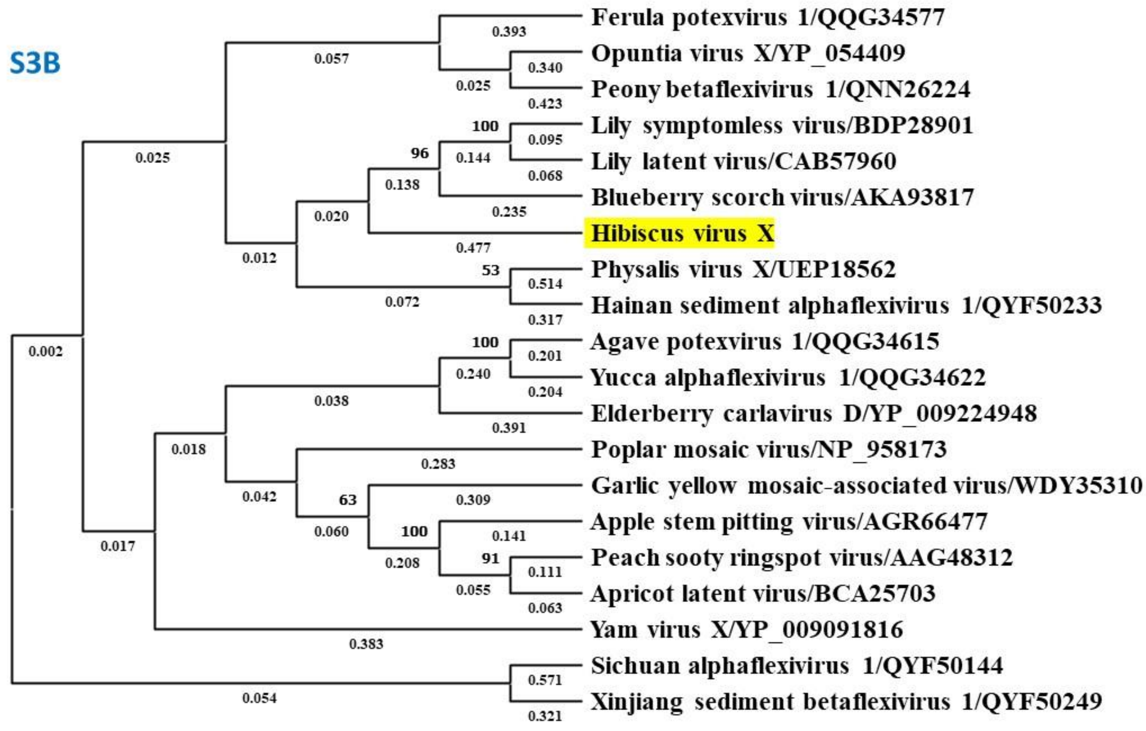

S3C

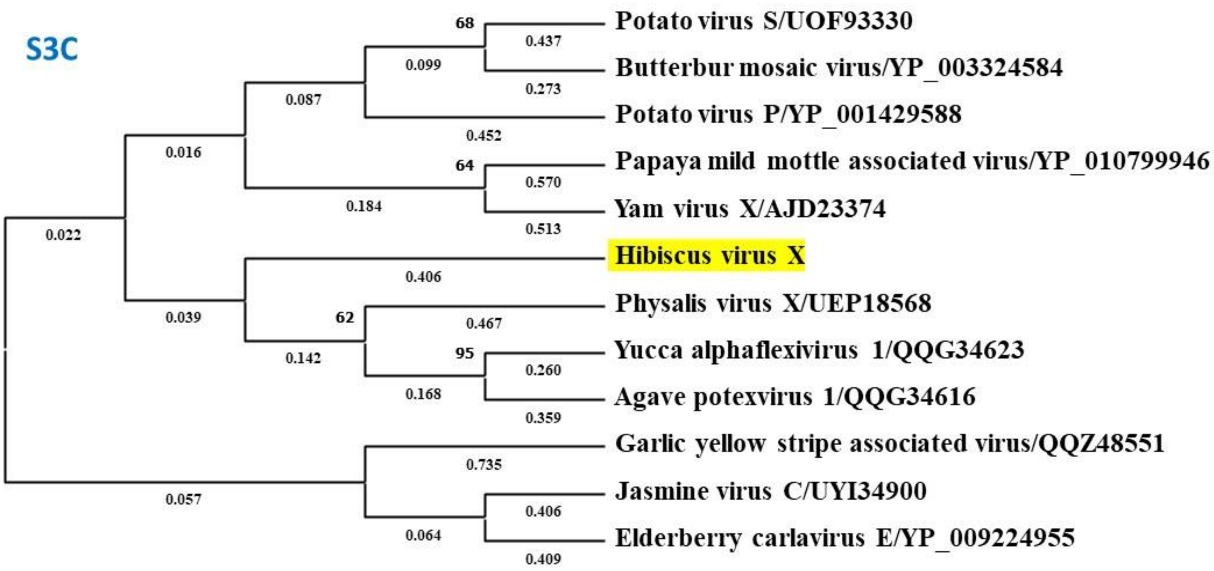

Potato virus S/UOF93330
Butterbur mosaic virus/YP_003324584
Potato virus P/YP_001429588
Papaya mild mottle associated virus/YP_010799946
Yam virus X/AJD23374
Hibiscus virus X
Physalis virus X/UEP18568
Yucca alphaflexivirus 1/QQG34623
Agave potexvirus 1/QQG34616
Garlic yellow stripe associated virus/QQZ48551
Jasmine virus C/UYI34900
Elderberry carlavirus E/YP_009224955

Supplementary Figure S4: Phylogenetic relationships of the hibiscus variant of physalis vein necrosis virus (PhyVNV) identified in the RsTHr high-throughput sequencing library with the 37 RNA1 (S4A) and 38 RNA2 (S4B) nepovirus species sequences (nucleotide level) utilizing the Multiple Alignment using Fast Fourier Transform (MAFFT, v7.520) program with bootstrap values of 1000 replicates

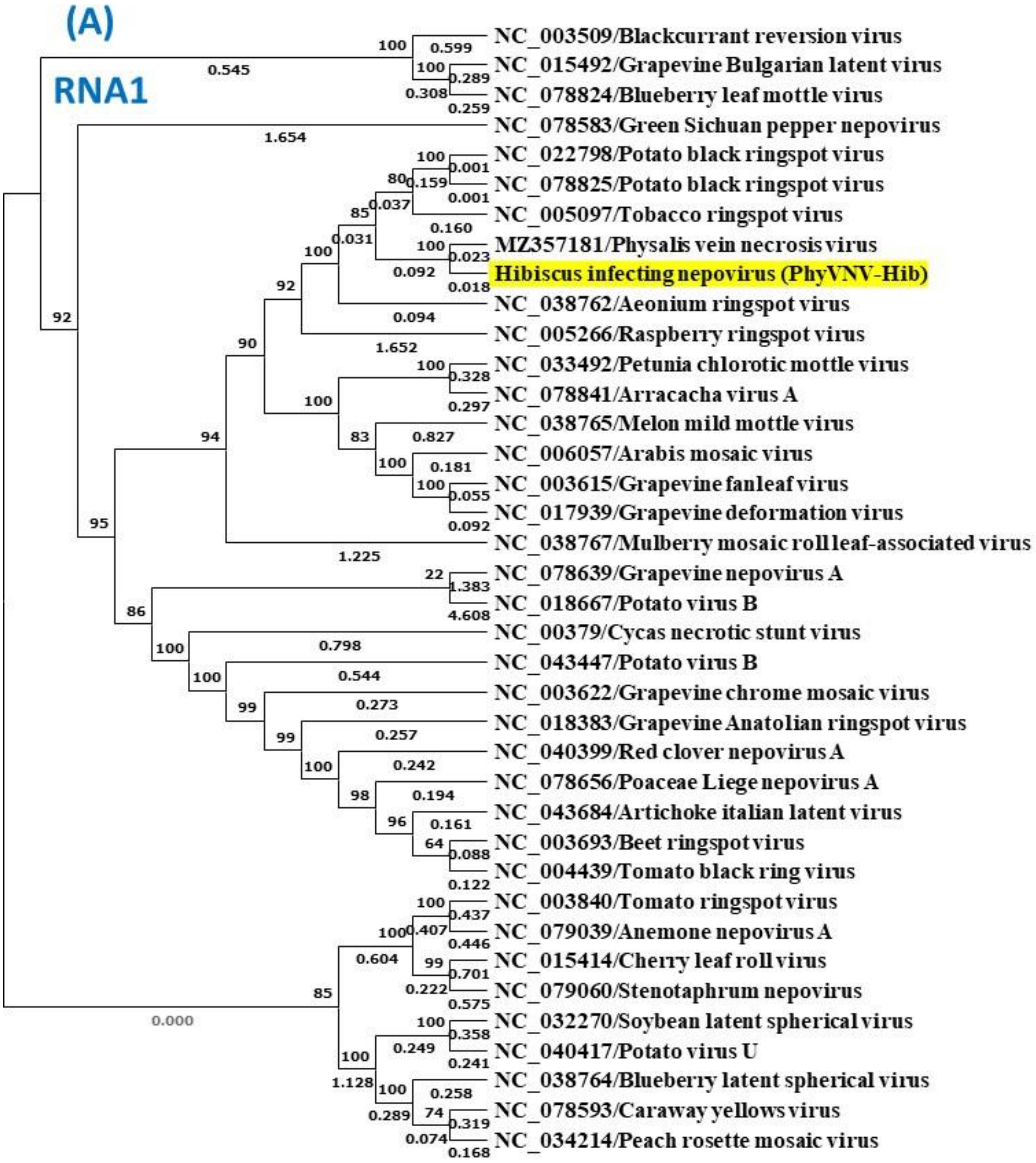

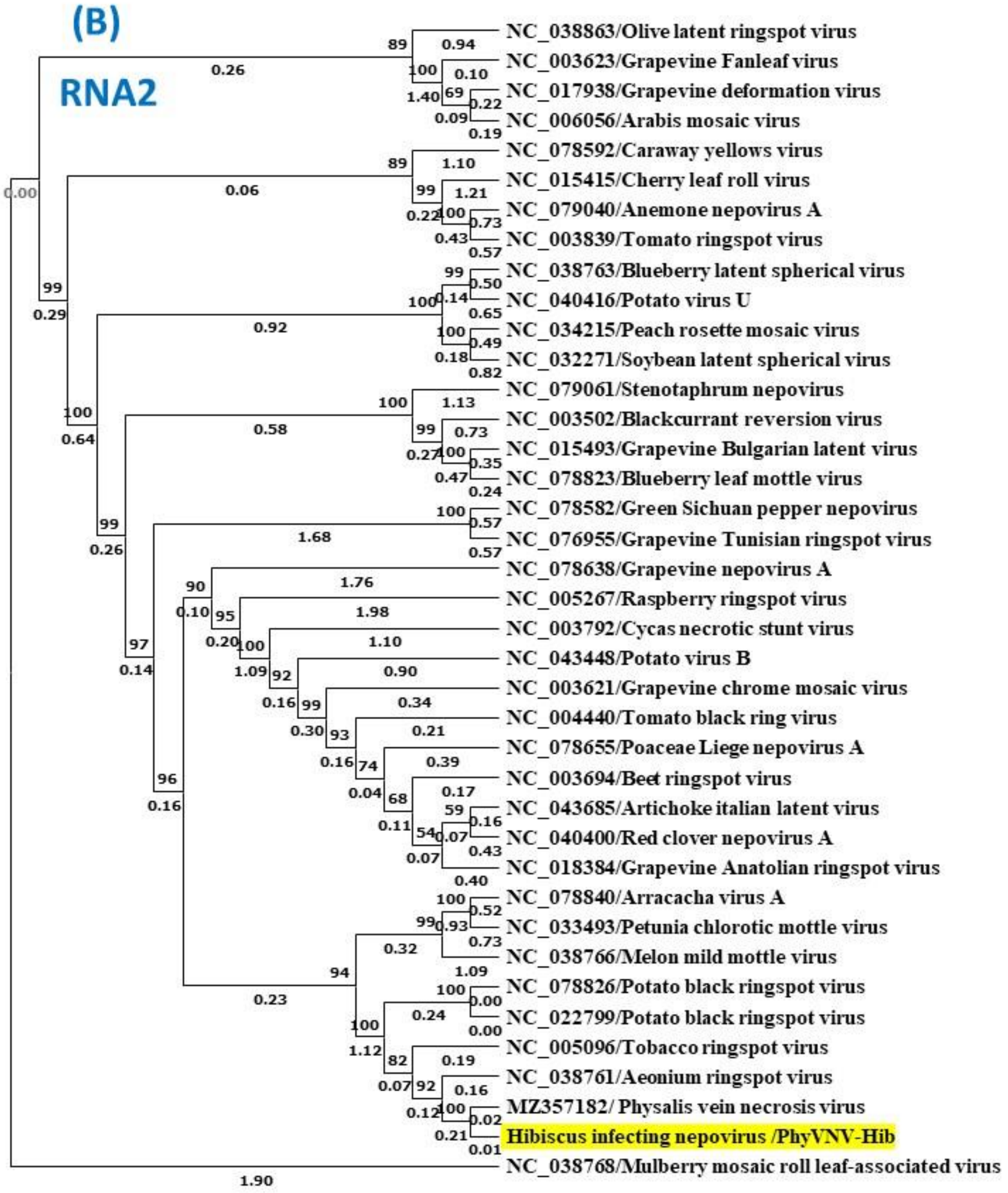
(B)
RNA2
NC_038863/Olive latent ringspot virus
NC_003623/Grapevine Fanleaf virus
NC_017938/Grapevine deformation virus
NC_006056/Arabis mosaic virus
NC_078592/Caraway yellows virus
NC_015415/Cherry leaf roll virus
NC_079040/Anemone nepovirus A
NC_003839/Tomato ringspot virus
NC_038763/Blueberry latent spherical virus
NC_040416/Potato virus U
NC_034215/Peach rosette mosaic virus
NC_032271/Soybean latent spherical virus
NC_079061/Stenotaphrum nepovirus
NC_003502/Blackcurrant reversion virus
NC_015493/Grapevine Bulgarian latent virus
NC_078823/Blueberry leaf mottle virus
NC_078582/Green Sichuan pepper nepovirus
NC_076955/Grapevine Tunisian ringspot virus
NC_078638/Grapevine nepovirus A
NC_005267/Raspberry ringspot virus
NC_003792/Cycas necrotic stunt virus
NC_043448/Potato virus B
NC_003621/Grapevine chrome mosaic virus
NC_004440/Tomato black ring virus
NC_078655/Poaceae Liege nepovirus A
NC_003694/Beet ringspot virus
NC_043685/Artichoke italian latent virus
NC_040400/Red clover nepovirus A
NC_018384/Grapevine Anatolian ringspot virus
NC_078840/Arracacha virus A
NC_033493/Petunia chlorotic mottle virus
NC_038766/Melon mild mottle virus
NC_078826/Potato black ringspot virus
NC_022799/Potato black ringspot virus
NC_005096/Tobacco ringspot virus
NC_038761/Aeonium ringspot virus
MZ357182/ Physalis vein necrosis virus
Hibiscus infecting nepovirus /PhyVNV-Hib
NC_038768/Mulberry mosaic roll leaf-associated virus

Supplementary Table S1: Published primer sequences of the citrus leprosis virus C2 and its hibiscus strain (CiLV-C2H), Hibiscus soymovirus (HSV) and Passion fruit green spot virus (PFGSV) are detailed with their citation.

Virus Name	Polarity	Primers		Amplified region	Amplicon size (Nts)	References
		Name	Sequence from 5' to 3'			
Citrus leprosis virus C2 (CiLV-C2)	sense	CiLV-C2-CPG-F	ATGAGTAACATTGTGTCGTTTTCGTTGT	Coat protein	795	Roy et al., 2013 [49]
	antisense	CiLV-C2-CPG-R	TCACTCTTCCTGTTCATCAACCTGTT			
Hibiscus strain of Citrus leprosis virus C2 (CiLV-C2H)	sense	R1CiLV-C2-GF5	TTGAAGTTYAAGTTGCAGGTTCAACG	RdRp and Coat protein	1447	Roy et al., 2018 [6]
	antisense	R1CiLV-C2H-R5	GGATGTTTACTCTCCCTGCTCTTCTG			
Hibiscus Soymovirus (HSV)	sense	HSV-REP-F	TAA CAG CAT TCA GTT GTC CAC CTC A	Replicase	631	Roy et al., 2023 [8]
	antisense	HSV-REP-R	TGA AGG ATT GCT CCC CAA GAA TCT T			
	sense	HSV-CPG-F	TAG ATC ACC ATC TCC AGT ACC TCC A	Coat protein	401	
	antisense	HSV-CPG-R	CTG TTT CTT GAC CAG TTG TTC CCA T			
	sense	SVF1	AGGAAGATGGTCGTTTTGGG	Transactivation	430	Wang et al. 2023 [31]
	antisense	SVR1	ACTGGACTGCTGGTTGTATG			
Passion fruit green spot virus (PFGSV)	sense	C13R	ATTCATGCGTTTCACGGTTA	RNA1	321	Ramos-González et al. 2020 [48]
	antisense	C13F	CGAATGCCTCTGACACAACT			
	sense	C6F	CGATATTTGATCAATCCGTT	RNA2	244	
	antisense	C6R	CACCTTAAAATTCGAGGGTT			
	sense	C8F	TTCATCGCAAGTTCGTATACCT		299	
	antisense	C8R	CTGTTGTGCCAAATCATCAA			

Supplementary Table S2: Analysis of thecomplete genome nucleotide sequence and the translated amino acid gene sequence of each open reading frame of the newly discovered Hibiscus carlaviruses with nine or ten nearest carlavirus relatives; percentage sequence coverage is indicated in parentheses. NB. Some of the related viruses listed in the table have multiple sequences in GenBank, thus the sequence accession numbers with the highest amino acid sequence identity for different HiCaV proteins may represent distinct isolates of the same virus. NS = not significant.

**Hibiscus Carlavirus**	**Nucleotide identity**									
**Full genome of HiCaVs**	**NC_018448**	**MW897314**	**LT898349**	**MW897296**	**MW371117**	**MZ173553**	**MK778784**	**MN059240**	**NC_077068**	**OM671312**
	**Sweet potato virus C6**	**Beijing sediment betaflexivirus**	**Elm carlavirus**	**Xinjiang sediment betaflexivirus 1**	**Cowpea mild mottle virus (MW201798)**	**Papaya chlorotic spot associated virus**	**Sweet potato chlorotic fleck virus**	**Garlic latent virus**	**Rose virus B**	**Ligustrum virus A**
Hibiscus carlavirus A (8229 nt)	68.70 (20)	67.37 (20)	66.67(18)	70.06 (16)	69.33 (15)	69.44 (15)	71.98 (10)	66.26 (14)	67.70 (19)	68.40 (15)
Hibiscus carlavirus B (8202)	68.77 (22)	67.67 (18)	66.73 (17)	70.42 (11)	71.22 (15)	70.79 (13)	67.62 (18)	67.11 (18)	70.86 (12)	67.43 (18)
Hibiscus carlavirus C (8117nt)	68.01 (22)	68.28 (18)	66.72 (17)	68.59 (16)	69.85 (22)	68.41 (20)	69.18 (18)	68.29 (15)	66.90 (26)	69.57 (13)
HiCaV-D (Contig 836 nt)	64.16 (45)	68.80 (14)	NS	72.34 (22)	70.87 (35)	71.52 (28)	73.54 (26)	72.31 (20)	71.49 (70)	73.31 (70)
HiCaV-D (Contig 3009 nt)	100 (01)	NS	69.53 (04)	NS	73.85 (02)	NS	74.79 (03)	68.27 (03)	77.55 (01)	82.86 (03)
HiCaV-D (Contig 641 nt)	68.75 (92)	67.83 997)	67.96 (56)	71.08 (88)	66.50 (92)	68.75 (56)	67.62 (92)	66.24 (84)	70.15 (68)	68.65 (57)
HiCaV-D (Contig 1899 nt)	69.66 (9)	75.44 (03)	NS	71.24 (08)	72.29 (12)	NS	70.92 (07)	69.30 (16)	70.59 (05)	65.35 (14)

**HiCaVs-ORF encoding gene**	**Amino Acid identity**								
**RdRp protein of HiCaVs**	**YP_006589919**	**BAL03071.1**	**YP_009224952.1**	**WCC72446.1**	**WCR76308.1**	**SNQ27851.1**	**WBG54312.1**	**WAK43334.1**	**QQX32728.1**
	**Sweet potato virus C6**	**Helleborus net necrosis virus**	**Elderberry carlavirus E**	**Rose virus B**	**American hop latent virus**	**Elm carlavirus**	**Poplar mosaic virus**	**Ligustrum virus A**	**Helenium virus S**
HiCaV-A	52.83 (81)	52.24 (79)	49.08 (79)	48.46 (80)	42.81 (99)	50.14 (75)	43.62 (99)	51.46 (78)	41.18 (99)
HiCaV-B	45.12 (100)	44.48 (99)	51.24 (79)	51.15 (78)	49.02 (80)	50.45 (79)	50.00 (80)	50.96 (78)	48.81 (79)
HiCaV-C	51.62 (83)	51.65 (80)	43.47 (100)	49.33 (78)	42.19 (99)	50.00 (76)	43.94 (99)	49.82 (80)	41.04 (99)
HiCaV-D (836 nt)	53.82 (98)	55.47 (98)	57.95 (58)	56.88 (99)	52.33 (98)	57.87 (90)	55.07 (99)	56.00 (98)	51.27 (98)
HiCaV-D (3009 nt)	40.99 (59)	38.64 (59)	35.79 (62)	39.82 (44)	36.67 (60)	36.48 (63)	37.94 (62)	35.34 (61)	34.69 (61)
HiCaV-D (641 nt)	69.95 (99)	72.77 (99)	66.20 (99)	64.79 (99)	65.26 (99)	67.61 (99)	69.95 (99)	65.73 (99)	66.20 (99)
**Coat protein of HiCaVs**	**YP_006589923**	**ACM45995.1**	**YP_009224950.1**	**YP_009224938.1**	**YP_009224932.1**	**YP_010802838.1**	**YP_003324585.1**	**WBG54313.1**	**QED43237.1**
	**Sweet potato virus C6**	**Helleborus net necrosis virus**	**Elderberry carlavirus D**	**Elderberry carlavirus B**	**Elderberry carlavirus A**	**Aconite virus A**	**Butterbur mosaic virus**	**Poplar mosaic virus**	**Garlic common latent virus**
HiCaV-A	48.64 (93)	50.17 (89)	49.80 (79)	48.76 (78)	48.39 (80)	44.98 (93)	49.03 (84)	42.72 (98)	44.74 (86)
HiCaV-B	52.14 (82)	50.52 (91)	49.39 (78)	47.76 (78)	46.77 (79)	46.69 (95)	49.23 (85)	48.57 (78)	47.64 (81)
HiCaV-C	46.84 (96)	47.39 (99)	50.20 (79)	48.76 (78)	47.98 (80)	50.21 (78)	47.97 (87)	43.56 (98)	48.96 (78)
HiCaV-D (last 444 nt of 1899 nt)	33.33 (68)	40.40 (66)	36.67 (60)	35.63 (58)	33.33 (62)	**No similarity**	37.96 (72)	31.29 (99)	42.53 (58)
**TGB1 protein of HiCaVs**	**QED43240.1**	**QED43588.1**	**SNQ27852.1**	**UZP17216.1**	**UDF48730.1**	**YP_010799376.1**	**YP_009224929.1**	**WMN13755.1**	**BAT32750**
	**Garlic common latent virus**	**Garlic latent virus**	**Elm carlavirus**	**Chrysanthemum virus B**	**Cowpea mild mottle virus**	**Birch carlavirus**	**Elderberry carlavirus A**	**Aconite virus A**	**Lily symptomless virus**
HiCaV-A	37.93 (100)	37.99 (99)	38.82 (100)	38.98 (100)	36.91 (98)	37.45 (99)	36.48 (100)	41.10 (100)	34.32 (100)
HiCaV-B	36.36 (100)	36.80 (100)	38.82 (100)	36.64 (100)	35.62 (98)	37.91 (88)	35.19 (100)	38.03 (100)	37.02 (100)
HiCaV-C	38.96 (100)	35.93 (100)	40.08 (100)	36.97 (100)	37.55 (98)	38.14 (99)	37.71 (100)	37.92 (100)	37.61 (100)
HiCaV-D	36.36 (100)	35.50 (100)	41.77 (100)	36.32 (100)	37.55 (98)	37.61 (88)	36.02 (100)	36.29 (100)	37.18 (100)
**TGB2 protein of HiCaVs**	**CAA92817.1**	**QED43133.1**	**BAZ96205.1**	**YP_003324583.1**	**YP_009664742.1**	**YP_006589921**	**AGR66477.1**	**SNQ27848.1**	**ACM45987.1**
	**Garlic latent virus**	**Garlic common latent virus**	**Shallot latent virus**	**Butterbur mosaic virus**	**Helleborus mosaic virus**	**Sweet potato virus C6**	**Apple stem pitting virus**	**Elm carlavirus**	**Helleborus net necrosis virus**
HiCaV-A	55.45 (100)	49.53 (100)	53.64 (100)	55.14 (100)	52.25 (100)	53.92 (95)	44.34 (96)	50.91 (100)	52.34 (95)
HiCaV-B	53.64 (100)	55.05 (100)	58.18 (100)	50.93 (100)	49.09 (100)	53.47 (94)	51.89 (96)	57.73 (89)	53.41 (82)
HiCaV-C	56.88 (100)	51.85 (100)	55.05 (100)	55.14 (100)	53.64 (100)	49.02 (95)	49.53 (96)	57.00 (92)	56.70 (88)
HiCaV-D	55.05 (100)	50.93 (100)	53.21 (100)	54.21 (100)	54.55 (100)	50.00 (95)	49.53 (96)	59.00 (92)	56.70 (88)
**TGB3 protein of HiCaVs**	**CAC83689.1**	**UCJ00265.1**	**QVD99728.1**	**QWT83588.1**	**ABD95728.1**	**QWT83669.1**	**YP_001429588.1**	**QTW21092.1**	**AAB81271.1**
	**Garlic latent virus**	**Cherry green ring mottle virus**	**Banmivirus BanMMV**	**Passiflora latent virus**	**Hydrangea chlorotic mottle virus**	**Potato virus S**	**Potato virus P**	**Potato virus X**	**Potato virus M**
HiCaV-A	35.48 (93)	36.84 (86)	32.26 (93)	40.62 (93)	40.62 (93)	42.37 (89)	48.57 (53)	30.16 (95)	54.84 (56)
HiCaV-B	45.59 (94)	38.81 (98)	30.88 (95)	32.76 (85)	42.86 (51)	40.38 (72)	38.81 (98)	42.59 (77)	33.33 (83)
HiCaV-C	39.68 (98)	35.43 (95)	38.71 (93)	33.85 (100)	39.68 (98)	38.71 (96)	39.06 (100)	40.00 (92)	37.93 (90)
HiCaV-D	40.32 (96)	35.70 (95)	39.39 (96)	34.92 (96)	40.62 (96)	38.71 (96)	48.57 (54)	40.35 (89)	58.06 (48)

Supplementary Table S3: Nucleotide and amino acid % identities and sequence coverage (in parentheses) of each ORF of the newly discovered hibiscus virus X with the seven nearest alphaflexivirus relatives and lily symptomless carlavirus. NB: TGB3 and CPG sequences for Sichuan alphaflexivirus 1 are not available in GenBank. Open reading frame numbers with nucleotide positions in the HiVX genome and the name of the gene representing each ORF with their lengths (number of amino acids) are shown. NS = not significant; ND = not determined, as these sequences are not available.

**Hibiscus Potexvirus**	**Nucleotide identity**							
**HiVX**	**Yucca alpha-flexivirus 1 (MW328742)**	**Agave potexvirus 1 (MW328740)**	**Physalis virus X (MZ357185)**	**Yam virus X (NC_025252)**	**Sichuan alpha-flexivirus 1 (MW897277)**	**Clover yellow mosaic virus (OK558678)**	**Strawberry mild yellow edge virus (MZ326676)**	**Lily symptom -less virus (LC720794)**
ORF1 (94-4170)	65.80 (42)	65.06 (55)	68.44 (16)	70.36 (16)	66.53 (48)	68.29 (21)	64.85 (38)	NS
ORF2 (4205-4870)	NS	NS	NS	84.38 (4)	NS	NS	NS	NS
ORF3 (4860-5252)	NS	NS	NS	NS	NS	NS	NS	73.68 (24)
ORF4 (5122-5316)	NS	NS	NS	NS	ND	NS	NS	NS
ORF5 (5355-6275)	66.01 (32)	69.59 (18)	NS	67.61 (15)	ND	60.08 (19)	NS	NS
Full Genome	65.80 (31)	65.06 (38)	68.44 (10)	70.36 (12)	66.53 (30)	68.29 (12)	64.85 (24)	73.68 (1)
**HiVX-ORF encoding gene**	**Amino Acid identity**							
	**Yucca alpha-flexivirus 1 (QQG34620-24)**	**Agave potexvirus 1 (QQG34613-17)**	**Physalis virus X (UEP18564-68)**	**Yam virus X (AJD3371-75)**	**Sichuan alpha-flexivirus 1 (QYF50142-44)**	**Clover yellow mosaic virus (UZP17132-36)**	**Strawberry mild yellow edge virus (UVT84557-61)**	**Lily symptomless virus (BAT32749-52)**
RdRp(1358)	55.10 (95)	54.26 (96)	49.27 (99)	49.77 (95)	58.51 (95)	50.36 (90)	48.20 (94)	23.36 (77)
TGB1 (221)	44.20 (100)	45.29 (100)	39.01 (98)	34.21 (99)	50.45 (99)	35.40 (99)	35.14 (97)	31.88 (100)
TGB2 (130)	41.67 (100)	41.22 (100)	35.66 (98)	47.96 (73)	53.23 (47)	42.00 (73)	45.26 (70)	47.37 (70)
TGB3 (64)	47.37 (87)	38.18 (85)	43.64 (85)	43.33 (90)	ND	NS	NS	37.74 (78)
CPG (306)	54.50 (68)	52.61 (68)	35.12 (66)	32.88 (69)	ND	32.94 (81)	34.64 (50)	29.10 (60)
